# Analysis of c.3499+200TA(7_56) and D7S523 Microsatellites Linked to Cystic Fibrosis Transmembrane Regulator

**Published:** 2012

**Authors:** Vahid Kholghi Oskooei, Mohammad Reza Esmaeili Dooki, Haleh Akhavan-Niaki

**Affiliations:** 1 *Cellular and Molecular Biology Research Center, Babol University of Medical Sciences, Babol, Iran. *; 2 *Non-Communicable Pediatric Diseases Research Center, Babol University of Medical Sciences, Babol, Iran.*

**Keywords:** Cystic Fibrosis, c.3499+200TA(7_56), D7S523, Iran

## Abstract

Cystic fibrosis (CF) is a life-limiting autosomal recessive disorder affecting principally respiratory and digestive system. It is caused by cystic fibrosis transmembrane conductance regulator (CFTR) gene mutation. The aim of this study was to determine the extent of repeat numbers and the degree of heterozygosity for c.3499+200TA(7_56) and D7S523 located in intron 17b and 1 cM proximal to the CFTR gene respectively. Both microsatellites were analyzed by direct electrophoresis of PCR product on 20% polyacrylamide gel in 40 Normal subjects and 40 CF patients originating from North Iran. 9 different alleles were found for D7S523 ranging from 16 to 24 repeats alleles. (CA)_20_ was the most prevalent allele both in normal individuals and CF patients with 21.3% and 20% frequencies respectively. Heterozygosity frequency of D7S523 in normal individuals and CF patients was 97.5% and 90% respectively. Eighteen different alleles were found for c.3499+200TA(7_56) ranging from 8 to 38 repeats alleles. (TA)_9 _was the most prevalent allele both in normal individuals and CF patients with 30% and 23.5% frequencies respectively. All normal subjects and 97.5% of CF patients showed heterozyous genotype. The high heterozygosity of the two studied microsatellites witnesses the dynamism of such markers. High degree of heterozygosity of c.3499+200TA(7_56) and D7S523 make these markers, a very useful tool for prenatal diagnosis especially in Iranian population.

Cystic fibrosis (CF) is the most common life-limiting autosomal recessive disorder in the white population. It is a complex multi-organ disease affecting respiratory and digestive system, male genital tract, and exocrine sweat glands (1). 1 in 2,000-3,000 Caucasian newborns are affected by CF and frequency of carriers is 1 in 26 (2). In Iranian population the incidence of CF was estimated to 1 in 6400 birth (3).

Mutation in cystic fibrosis transmembrane conductance regulator (CFTR), located on the long arm of chromosome 7 (7q21-34) causes CF (-). CFTR span approximately 150 kb of genomic DNA, consisting of 27 exons and encodes a mature 6.5 kb mRNA transcript (7-8). CFTR protein consists of 1480 amino acids and forms a chloride channel, an essential component of epithelial chloride transport systems in many organs, including the intestines, pancreas, lungs, sweat glands, and kidneys (9-10).

More than 1800 CFTR mutation and polymorphisms have been identified (11). The vast majority of mutations have frequencies less than 0.1% and their distribution depend on race and/or ethnicity (12). The most common disease-causing mutation, p.Phe508del, is found in some 70% of Canadian, American and Northern European Caucasian patients (13). There is a decrease in frequency from European to Middle East countries where this allele represent about 18% to 24% of mutant alleles (3, 14-15).

Although molecular diagnosis is the unique approach to perform prenatal diagnosis in at risk couples, there is no guarantee for detection of the two disease-causing mutations by direct gene analysis procedures (16). In other hand, in countries like Iran, high heterogeneity of CFTR mutations, make molecular diagnosis conditions more complicated (17).

Segregation analysis of CFTR polymorphic markers can be an efficient alternative in families presenting a previous history of the disease with one or two unknown mutations. This method not only reduces errors due to methods of mutation detection, maternal contamination or human manipulations, but also can be used to search for possible associations between haplotypes and CF mutations (16).

c.3499+200TA(7_56) is a highly polymorphic microsatellite containing TA-repeats located in intron 17b of CFTR gene and have been shown to have at least 24 different alleles with sizes ranging from 7 to 56 repeats (18). D7S523 is a microsatellite containing CA-repeats located 1 cM proximal to the CFTR gene which was reported to show 80% heterozygote frequency in Europe (19). In this report we considered c.3499+200TA(7_56) and D7S523 polymorphisms on CFTR gene in normal individuals and cystic fibrosis patients in North Iran.

## Materials and methods

**Patients**

40 normal adult fertile males or females and 40 CF patients under age 14 and presenting pulmonary complications and elevated sweat chloride values (> 60 mEq/L) were studied. All subjects were from the North of Iran. Genomic DNA was extracted from peripheral blood using Alkalin lysis method.

**Molecular analysis**

**Fig 1:**
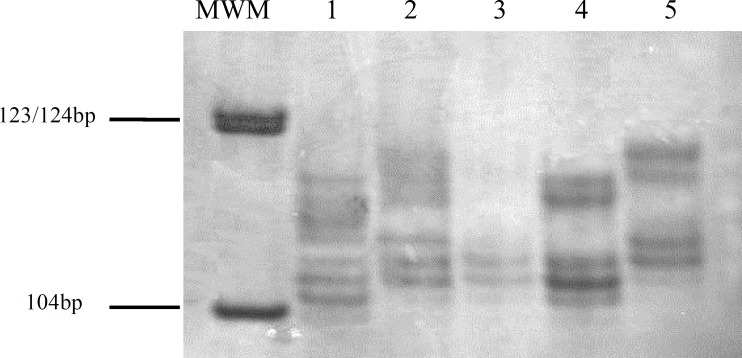
Separation of PCR products of D7S523 on a 20% polyacrylamide gel. MWM: Molecular weight marker V (Roche, Germany

D7S523 polymorphism was analyzed by direct elecctrophoresis of PCR product on 20% polyacrylamide gel. PCR amplifications were carried out in 25 µl volume reaction containing 250 µM dNTPs, 1.5 mM MgCl_2_, 0.2 pM each forward and reverse primers, 0.5 unit Taq DNA polymerase. Primers sequence is presented in Table 1. The PCR conditions were as follows: denaturation at 94ºC for 4 minutes, then 35 cycles, consisting of 30 second denaturation at 94ºC, annealing at 55ºC for 30 seconds and extension at 72ºC for 30 seconds, followed by final extension at 72ºC for 10 minutes. Amplification of expected fragment was confirmed by sequencing of one PCR product. Figure 1 shows a representative result of genotyping for D7S523 locus. Although we found more than 2 bands (up to 6 bands) for each PCR reaction, we presumed that lower bands correspond to homoduplexes and higher bands correspond to heteroduplexes. Repeat numbers were verified for at least one of the patients by performing Sanger dideoxy sequencing analysis (Bioneer, South Korea). 

Analysis of c.3499+200TA(7_56) was performed upon nested-PCR. Primary PCR was carried out by external primer in 25 µl volume reaction containing 250 µM dNTPs, 1.5 mM MgCl_2_, 0.2 pM each forward and reverse primers, 0.5 unit Taq DNA polymerase. Thermo-cycling conditions was initial denaturation at 94°C for 4 minutes, followed by 30 cycles of denaturation at 94°C for 30 seconds, annealing at 60°C for 30 seconds, extension at 72°C for 30 seconds. Cycling culminated with a final extension at 72°C for 4 minutes. For the secondary PCR, first round PCR products were used as template and internal forward and reverse primers were used. Final PCR products were separated on a 20% polyacrylamide gel. Table 1 shows the sequences of external and internal primers used to amplify c.3499+200TA (7_-_56).

**Table 1:** Primers used for analysis of D7S523 and c.3499+200TA(7_56) in CFTR gene

**Locus **	**Primer sequences 5** **′** **→3** **′**
D7S523:	F: TGTGGAAAAATATTCTGGGAAGA
R: ACCTGTTGACATTTTTAAAACCA	
c.3499+200TA(7_-_56):	
External primers	F: GCTGCATTCTATAGGTTATC
R: AAACTTACCGACAAGAGGAA	
Internal primers	F: CAAATAATTTCCTTGAAATCGGATA
R: TTAAAACTGTGAAAACAGGGATAAT	
As c.3499+200TA(7-56) was amplified by nested PCR, 2 sets of primers (External and Internal) were used to analyze this microsatellite	

## Results

**D7S523 analysis**

**Fig 2:**
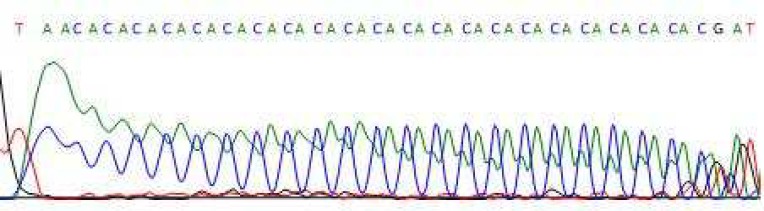
Sequence analysis of D7S523 locus: Sequencing data for a CF patient shows a CA_(23)_/CA_(22)_ genotype

Nine different alleles were found in studied population ranging from 16 to 24 repeats. (CA)_20_ was the most prevalent allele both in normal individuals and CF patients with 21.3% and 20% frequencies respectively. (CA)_16_ and (CA)_24 _alleles had the lowest prevalence in normal individuals with 1.3% and in CF patients with 2.5% frequency. (CA)_21_ /(CA)_20_ genotype was the most frequent both in normal individuals and CF patients with 22.5% abundance. Heterozygosity frequency of D7S523 in normal individuals and CF patients was 97.5% and 90% respectively. The distribution of different D7S523 alleles is represented in Table 2. Figure 2 shows a sequencing result for a (CA)_23_ /(CA)_22 _patient.

**c.3369+213TA(7_56)** **analysis**

Eighteen different alleles were found in population ranging from 8 to 38 repeats alleles. (TA)_9 _was the most prevalent allele both in normal individuals and CF patients with 30% and 23.5% frequencies respectively. (TA)_9 _/(TA)_8 _ genotype was the most prevalent genotype in normal individuals and CF patients with 52.5% and 35%% frequencies. All normal subjects and 97.5% of CF patients showed heterozyous genotype. Allelic distribution of c.3499+200TA(7_56) locus is represented in Table 2.

**Table 2:** Allele frequencies of c.3499+200TA(7_56)and D7S523

** CF (n = 80)**		** Normal (n = 80)**		** No. of repeats**	
** CA**	**TA **	** CA**	**TA **	**CA**	**TA **
-	14(17.5%)	1(1.3%)	21(26.3%)	16	8
3(3.8%)	19(23.5%)	4(5%)	24(30%)	17	9
11(13.8%)	6(7.5%)	4(5%)	3(3.8%)	1800	10
12(15%)	1(1.3%)	15(18.8%)	-	19	11
16(20%)	-	17(21.3%)	1(1.3%)	20	24
15(18.8%)	-	15(18.8%)	1(1.3%)	21	25
13(16.3%)	-	9(11.3%)	2(2.5%)	22	27
8(10%)	-	7(8.8%)	3(3.8%)	23	28
2(2.5%)	-	-	3(3.8%)	24	29
	1(1.3%)		2(2.5%)		30
	4(5%)		2(2.5%)		31
	7(8.8%)		6(7.5%)		32
	8(10%)		6(7.5%)		33
	10(12.4%)		3(3.8%)		34
	6(7.5%)		2(2.5%)		35
	2(2.5%)		1(1.3%)		36
	1(1.3%)		-		37
	1(1.3%)		-		38

**Table 3:** Genotype frequencies of c.3499+200TA(7_56)and D7S523

**CF patients (n = 80)**		**Normal (n = 80)**		**Genotypes of **	
**CA**	**TA **	**CA**	**TA **	**CA**	**TA **
	14(35%)	1(2.5%)	21/(52.5%)	17/16	9/8
3(7.5%)	5(12.5%)	3(7.5%)	3(7.5%)	18/17	10/9
5(12.5%)	1(2.5%)	7(17.5%)	-	19/18	11/10
3(7.5%)	-	6(15%)	1(2.5%)	20/19	25/24
1(2.5%)	-	2(5%)	1(2.5%)	20/18	28/27
9(22.5%)	-	9(22.5%)	1(2.5%)	21/20	29/28
1(2.5%)	-	2(5%)	2(5%)	21/19	30/29
6(15%)	1(2.5%)	2(5%)	-	22/21	31/30
4(10%)	3(7.5%)	5(12.5%)	2(2.5%)	23/22	32/31
2(5%)	2(5%)	2(5%)	2(2.5%)	24/23	33/32
1(2.5%)	6(15%)	-	2(5%)	18/18	34/33
1(2.5%)	4(10%)	-	2(2.5%)	19/19	35/34
1(2.5%)	2(5%)	-	-	20/20	36/35
-	1(2.5%)	1(2.5%)	-	21/21	38/37
1(2.5%)	1(2.5%)	-	-	22/22	32/32
1(2.5%)	-	-		23/23	-

## Discussion

Analysis of D7S523 microsatellite showed that (CA)_20_ is the most common allele in both CF patients and normal subjects with 20% and 21.3% frequencies, respectively. In similar studies in Caucasian population, D7S523 microsatellite repeats varied between 1 to 7 (20), while in the present study, CA repeats number varied between 16 to 24. This difference can be due to unequal crossing over or replication slippage. Allelic variation of D7S523 locus linked to F508del mutation demonstrates that this locus is highly unstable (20). (CA)_21_ /(CA)_20_ genotype was the most frequent genotype in both normal individuals and CF patients. Heterozygosity frequency of D7S523 in normal individuals and CF patients was higher than 90%. Such a high heterozygocity make this locus a suitable one for CFTR alleles tracking by linkage analysis of families with previous history of the disease and no obvious mutant allele recognized by routine mutation detection methods.

c.3499+200TA(7_56) survey showed that (TA)_9 _allele was the most prevalent allele in normal individuals and CF patients with 30% and 23.5% frequencies, respectively. In similar studies of European CF patients, (TA)_31_ was the most frequent allele (21-22)_._ This divergence could be related to different spectrum and abundance of CFTR mutations of Iranian patients in comparison to European CF patients. c.3499+200TA(7_56) was highly polymorphic in the present study as all normal subjects and 97.5% of CF patients were heterozygous at this locus. Similar studies in Caucasian populations also reported a high degree of heterozigosity for this locus (22-24).

Despite the relatively important degree of consanguinity (around 40%) in both normal subjects and CF patients (25), the heterozygosity of the two studied microsatellites was higher than 80% witnessing the dynamism of such markers. High degree of heterozygosity of c.3499+200TA(7_56) and D7S523 make these markers, a very useful tool for prenatal diagnosis especially in Iranian population where CFTR mutations are very heterogeneous. Moreover heterozygosity of polymorphic microsatellites can aid in detecting possible maternal or exogenous DNA contaminations during prenatal diagnosis of genetic disorders.
